# Quadrivalent Influenza Vaccine-Induced Antibody Response and Influencing Determinants in Patients ≥ 55 Years of Age in the 2018/2019 Season

**DOI:** 10.3390/ijerph16224489

**Published:** 2019-11-14

**Authors:** Maria Ganczak, Paulina Dubiel, Marzena Drozd-Dąbrowska, Ewelina Hallmann-Szelińska, Karol Szymański, Lidia B. Brydak

**Affiliations:** 1 Department of Infectious Diseases, University of Zielona Góra, Zyty 28, 65-046 Zielona Góra, Poland; 2 Department of Epidemiology and Management. Pomeranian Medical University, Zolnierska 48, 71-210 Szczecin, Poland; 3 Department of Influenza Research, National Influenza Center, National Institute of Public Health–National Institute of Hygiene, Chocimska 24, 00-791 Warsaw, Poland

**Keywords:** influenza, vaccination, quadrivalent influenza vaccine, QIV, immunogenicity, elderly

## Abstract

The effects of immunization with subunit inactivated quadrivalent influenza vaccine (QIV) are not generally well assessed in the elderly Polish population. Therefore, this study evaluated vaccine-induced antibody response and its determinants. Methods: Consecutive patients ≥ 55 years old, attending a Primary Care Clinic in Gryfino, Poland, received QIV (A/Michigan/ 45/2015(H1N1)pdm09, A/Singapore/INFIMH-16-0019/2016 (H3N2), B/Colorado/06/2017, B/Phuket/ 3073/2013) between October-December 2018. Hemagglutination inhibition assays measured antibody response to vaccine strains from pre/postvaccination serum samples. Geometric mean titer ratio (GMTR), protection rate (PR) and seroconversion rate (SR) were also calculated. Results: For 108 patients (54.6% males, mean age: 66.7 years) the highest GMTR (61.5-fold) was observed for A/H3N2/, then B/Colorado/06/2017 (10.3-fold), A/H1N1/pdm09 (8.4-fold) and B/Phuket/ 3073/2013 (3.0-fold). Most patients had post-vaccination protection for A/H3N2/ and B/Phuket/3073/ 2013 (64.8% and 70.4%, respectively); lower PRs were observed for A/H1N1/pdm09 (41.8%) and B/Colorado/06/ 2017 (57.4%). The SRs for A/H3N2/, A/H1N1/pdm09, B Victoria and B Yamagata were 64.8%, 38.0%, 46.8%, and 48.2%, respectively. Patients who received QIV vaccination in the previous season presented lower (*p* < 0.001 and *p* = 0.03, respectively) response to B Victoria and B Yamagata. Conclusions: QIV was immunogenic against the additional B lineage strain (B Victoria) without significantly compromising the immunogenicity of the other three vaccine strains, therefore, adding a second B lineage strain in QIV could broaden protection against influenza B infection in this age group. As the QIV immunogenicity differed regarding the four antigens, formulation adjustments to increase the antigen concentration of the serotypes that have lower immunogenicity could increase effectiveness. Prior season vaccination was associated with lower antibody response to a new vaccine, although not consistent through the vaccine strains.

## 1. Introduction

Influenza is a contagious, acute respiratory disease, usually caused by Influenza A or B viruses, with seasonal infections that can lead to numerous complications, hospitalization and even death. Children under the age of 5, adults over 50 years of age, pregnant women and those with certain chronic medical conditions are most at risk [1,2,3,4,5].

According to the World Health Organization (WHO) influenza occurs globally, with the annual attack rate estimated at 5–10% in adults and 20–30% in children; there are about 290,000–650,000 deaths reported annually [6,7]. In Poland, The Department of Influenza Research, the National Influenza Center at the National Institute of Public Health—The National Institute of Hygiene (NIPH-NIH) collects and publishes virological and epidemiological data on incidences and suspected cases of influenza. The incidence of influenza and influenza-like illness has been increasing over the past decade. A rise in the number of referrals for hospitalization, due to influenza and post-influenza complications has also been noted. In the 2017/2018 season, the number of cases and suspected cases of influenza in Poland was 5,337,619 (10% more than the previous season), with 48 deaths reported. These could be due to the B-strain divergence. The high peak season had an average daily incidence exceeding 100/100,000 of the population [8]. Of note, according to the NIPH-NIH, although the number of cases decreased in the following season, 147 casualties were reported between 1 October 2018 to 31 April 2019 [8].

The US Centers for Disease Control and Prevention and CDC’s Advisory Committee on Immunization Practices recommended annual influenza vaccination for all persons aged ≥6 months who do not have contraindications [5]. Although influenza vaccines are not the most effective when compared to other types of vaccines, especially in the elderly, they are still the cheapest and most effective way to prevent infections and complications caused by influenza and are vital for individuals at high-risk of serious post-influenza complications [5]. An additional, supportive strategy with accumulating evidence is the extra protection of non-immune high-risk persons with an increase in immunity among the vaccinated and healthier individuals; this prevents the circulation of influenza in the community (the vaccine herd effect) [9].

Vaccinations against influenza are still neglected, and the vaccination rates remain low worldwide. According to the WHO, influenza vaccination uptake in Poland is in one of the lowest in Europe (3.6%). In patients with chronic diseases, as well as the elderly, immunization coverage is higher than in the general population; however, this still remains well below the recommended level, which is the vaccination of 75% of the key risk groups [6,10,11,12]. As an example, Nitzch-Osuch et al. found the following influenza vaccination coverage in the respective groups of Polish patients with chronic diseases—58% in pulmonary, 34% in hemodialyzed, 32% in cardiovascular and 9% in thyroid cancer patients [10]. With regards to the elderly, uptake is around 35%, the highest reported among those living in urban areas, well-educated regarding the influenza vaccination, having a vaccinated family member and immunized in the previous season(s) [11]. 

Two types of influenza vaccine are available, an inactivated preparation and an attenuated influenza vaccine. The inactivated vaccines come in three major formulations: Whole-virus, split virus or subunit vaccines, prepared from embryonated chicken eggs, inoculated individually with each virus type. The whole-virus vaccine is prepared from harvested allantoic fluid, chemically inactivated and subsequently purified to remove non-viral protein contaminants. In the split virus vaccine, the virus is disrupted by a detergent. In subunit vaccines, hemagglutinin (HA) and neuraminidase (NA) are further purified by the removal of other viral components. Live, attenuated influenza vaccines are based on temperature-sensitive variant vaccine virus strains that replicate well in the nasopharynx, but poorly in the lower respiratory tract [13]. 

For conventional influenza vaccines to be maximally effective, the vaccine viruses have to be antigenically matched to the influenza viruses circulating in humans [13]. In the last decade, two influenza A subtypes (H1N1 and H3N2) have predominated in Europe and worldwide, but influenza B viruses have recently become increasingly prominent [14]. In the 1970s, influenza B viruses diverged into two major antigenically distinct lineages, B/Victoria and B/Yamagata [15]. Since then, these two genetic lineages have co-circulated, which makes it difficult to predict which one will pre-dominate next season [14,15]. The most widely used seasonal influenza vaccine was the trivalent inactivated vaccine (TIV), composed of the three seasonal influenza virus strains currently circulating: Two influenza A virus types (H3N2 and H1N1), but only one B lineage (either B/Victoria or B/Yamagata). However, the B-lineage strain in TIVs and the dominant circulating B-lineage strain have differed in about 25% of the influenza season [16]. To reduce the chance of vaccine mismatch, a quadrivalent influenza vaccine (OIV) has been recently approved that includes an additional type B strain to represent both antigenic lineages [13,17,18]. Its immunogenicity and a safety profile, comparable to those of TIV, have the potential to overcome the drawbacks of erroneously predicting which B lineage will predominate in a given year [19,20,21]. Quadrivalent influenza vaccine (QIV) is expected to provide significant public health and economic benefit, as shown in recent studies [21].

The WHO recommended QIV for the northern hemisphere for the 2018/2019 influenza epidemic season which consists of A/Michigan/45/2015 (H1N1)pdm09-like virus, A/Singapore/ INFIMH-16-0019/2016 (H3N2)-like virus, B/Colorado/06/2017-like virus (Victoria lineage) and B/Phuket/3073/ 2013-like virus (Yamagata lineage) [22]. Influenza vaccination with QIV was recommended for 2018/2019 season in the Polish National Immunization Program for all citizens aged >55 years [23].

One commonly accepted approach is the measurement of influenza-specific antibody titers as a correlate of protection. Titers are traditionally measured using a hemagglutination inhibition (HAI) assay, which quantifies the ability of hemagglutinin (HA)-specific antibodies to block the N-acetyl-neuraminic acid-mediated viral agglutination of red blood cells [24,25]. Using the set guidelines of this assay, vaccine immunogenicity can be measured based on HAI antibody titers obtained on day ≥28. Parameters commonly used to expresses seroresponse to influenza vaccination are mean fold increase, seroprotection and seroconversion rates [26,27,28]. While discrepancies can be found in surveys focusing on antibody response to influenza vaccine in the elderly, a quantitative review concluded that host-related factors, such as gender, BMI, preexisting immunity, genetic polymorphisms, and the presence of chronic underlying conditions could compromise influenza vaccine responsiveness [29,30]. HAI antibodies are significantly lower in older adults who were vaccinated, than compared to younger adults [30]. Although limited data exist, gender-differences have also been reported in response to diverse influenza vaccines with females having greater antibody responses than males following vaccination [29]. Obesity has also been associated with a decreased immune response to influenza vaccination. In addition, several studies document the influence of host genetic background on the immune response to influenza vaccination [29]. There is also a correlation between health status in older adults and HAI titers, i.e., healthy individuals having significantly higher levels of titers than those with chronic diseases [29,31].

There are methodological discrepancies among the meta-analyses of seasonal influenza vaccines efficacy and effectiveness for the elderly [32]. Although most vaccines show statistically significant efficacy, this is within a highly variable range [32,33]. The results of the measurement of influenza-specific antibody titers have been described previously; however, these mainly referred to older adults from the US and Western Europe [16,19,20]. Polish data on QIV immunogenicity in this age group are lacking. Therefore, the objective of this study was to evaluate the immunogenicity of a subunit inactivated QIV vaccine in Polish adults ≥55 years of age.

## 2. Materials and Methods

### 2.1. Setting, Study Population, and Sampling

The study was conducted among consecutive patients reporting to the primary care clinic (PCC) in Gryfino, Poland, in the vaccination season between October 2018–January 2019. The study group consisted of consecutive patients vaccinated with a QIV recommended by the WHO for that season. Inclusion criteria: Age ≥ 55 years, lack of co-existing diseases that could affect the cognitive functions of the subject, lack of contraindications to vaccination and informed written consent. Participation was voluntary. 

### 2.2. Vaccine and Vaccination

Subunit, inactivated QIV (Abbott Biologicals, Olst, The Netherlands) was provided in prefilled syringes and administered by injection intramuscularly, using a 19 mm needle into the deltoid muscle to all subjects during the 2018–2019 influenza season. The cold chain was preserved, and the vaccines were stored at 2–8 ℃. A standard dose of QIV (0.5 mL) contained 15 µg of hemagglutinin per strain (total HA concentration of 60 μg): A/Michigan/45/2015 (H1N1)pdm09-like strain (A/Singapore/GP1908/ 2015, IVR-180), A/Singapore/INFIMH-16-0019/2016 (H3N2)-like strain (A/Singapore/INFIMH-16-0019/2016, NIB-104), B/Colorado/06/2017-like strain (B/Victoria/2/87 lineage) (B/Maryland/15/2016, NYMC BX-69A) and B/Phuket/3073/2013-like strain (B/Yamagata/16/88 lineage) (B/Phuket/3073/ 2013, wild type).

### 2.3. Serological Testing

Blood samples were collected twice, once before vaccination and four weeks after. Samples (1 mL) were centrifugated (15 min/4500 r.p.m.), stored at ≤−20 °C and then transported to the laboratory at the Department of Influenza Research (National Influenza Center, the NIPH-NIH) in Warsaw where they were tested. Briefly, sera were inactivated to remove non-specific hemagglutination inhibitors, which may affect a false positive result in an HAI test. Therefore, sera were treated with Receptor Destroying Enzyme (RDE), obtained from the Vibrio cholerae cell filtrate, and incubated at +37 °C. The test consisted of determining antibody titers (anti-HA) in serum by means of an HAI using a 0.75% solution of turkey red blood cells and reference strains of influenza virus, multiplied in chicken embryos, according to WHO recommendations [34]. 

Each study participant was given a code number, also placed both on the questionnaire and on a test tube. On 31 January 2019, participants were able to obtain information about their before/after vaccination serological tests results.

### 2.4. Vaccination Immunogenicity Assessment

On the basis of the results obtained after sero-testing, relevant parameters were calculated to assess the immunogenicity of a QIV. The current study assessed QIV-induced HAI antibody geometric mean titers (GMTs), seroconversion and seroprotection rates. Humoral responses were assessed on the basis of guidelines developed by the Committee for Proprietary Medicinal Products (CPMP) and the European Agency for the Evaluation of Medicinal Products (EMEA, now the European Medicine Agency, EMA) [1,26]. The following parameters were evaluated:

- GMT (geometric mean titers) calculated at baseline (day 0) and 28–36 days after vaccination,
- Average increase in antibody titers: GMTR (geometric mean titers ratio)—geometric mean of the individual post-vaccination/pre-vaccination titer ratios,
- PR (protection rate)—the proportion of subjects with an HAI antibody titer ≥ 1:40,

For the purpose of this study, the widely accepted HAI antibody titer of at least 1:40 was used to define seroprotection [16,35,36]. However, there is an ongoing debate whether this definition and serum antibody titers, in general, are valid correlates of protection [37,38,39,40,41]. According to Greenberg [36] and Chang [16] who evaluated the immunogenicity of a QIV in independent RCTs, as well as to current US guidelines [42], the HAI antibody titer remains an acceptable surrogate marker that is likely to predict clinical benefit.

- Seroconversion—either (1) an HAI titer < 10 at day 0 and a post-vaccination (day 28–36) HAI titer ≥ 40 or (2) an HAI titer ≥10 at day 0 and a ≥4-fold increase in HAI titer between day 0 and post-vaccination [16].

Vaccination response in individuals aged >60 years is confirmed as effective when GMTR ≥ 2.0, PR ≥ 60%, SR ≥ 30% [26,31].

### 2.5. Ethical Approval

The project received consent from the Bioethical Committee of Pomeranian Medical University in Szczecin (KB-0012/109/18).

### 2.6. Statistical Analysis 

Data were analyzed using a customized program STATISTI-CA PL, Version 12.5 (StatSoft, Kraków, Poland). Categorical data were presented as frequencies with percentages and continuous data as means. The primary endpoints, HAI antibody titer, GMTR, seroconversion and seroprotection rate, were analyzed. HAI antibody titers were analyzed using geometric mean titer (GMT) and geometric standard deviations. GMTR, seroprotection and seroconversion were defined as in 2.4 sub-section. Protective HAI antibody titers before vaccination in the 2018/2019 season by the previous season vaccination were compared using the Fisher exact test.

Regarding determinants influencing seroconversion categorical (binary) variables (such as age: Up to 67/ ≤ 67 years; gender: Male/female; BMI: < 25/ ≥ 25 kg/m^2^; smoking: Yes/no; alcohol consumption: Yes/no; chronic diseases: Yes/no; the self-reported occurrence of symptoms of upper respiratory tract infection in the current epidemic season: Yes/no; previous influenza vaccinations: Yes/no; vaccination in the previous season: Yes/no) groups were compared using the Fisher exact test. 

A *p*-value was statistically significant if ≤0.05. 

## 3. Results

### 3.1. Characteristics of Study Participants

Of 121 patients invited to participate, 108 (89.3%) agreed (mean age: 66.7 years, SD 6.7; range: 55–85 years), 54.6% were males; Table 1. Regarding BMI, 39.8% were overweight, and 38.0% were obese. Smoking at the time of vaccination was declared by 18.5% participants, 41.7% had quit smoking, and 39.8% declared that they had never smoked. More than half of the respondents (57.4%) declared alcohol consumption 1–2 times a month, 16.7% never consumed alcohol, 16.7% had drunk in the past. The vast majority of respondents (83.3%) had not had respiratory symptoms during the current season, 13.0% self-reported respiratory symptoms and 3.7% did not know. About one-third of patients (34.3%) reported comorbidities, mainly diabetes (25.0%), followed by cancers (7.4%), autoimmune diseases (4.6%) and renal failure (3.7%). 

### 3.2. Influenza Vaccination

About two-thirds of the participants (63.0%) had never been vaccinated against influenza, 13.9% were vaccinated only once in their lifetime, 23.2%—more than once. Only 15.7% of respondents reported being vaccinated in the previous season; their vaccination records showed they were vaccinated with QIV.

### 3.3. Serologic Antibody Response after Influenza Vaccination 

Paired pre- and ≥28 days (28–36 days) post-vaccination sera were available from 108 vaccinated patients. Serologic antibody response after QIV vaccination in terms of GMT, GMTR, PR and SR are presented in Table 2.

HAI antibody titers at baseline were the highest for B Yamagata lineage strain and similar regarding the rest studied strains (Table 2). Immunization with a QIV increased HAI antibody titers by 62-fold against the A/H3N2/ strain and by 3.0 to 10.3-fold against the B strains. Post-vaccination PRs were 42–65% against the A strains and 57% to 70% against the A/H1N1/pdm09 and B strains, and SRs occurred in 38–65% of participants for the A strains and in 47% to 48% for the B strains.

In detail, no protection against A/H3N2/ was observed in the study group before vaccination (PR 0.0%), however, the percentage of participants with antibody titers ≥ 1:40 increased significantly (PR 64.8%) after immunization and the proportion of participants with seroconversion also equaled 64.8%; the GMTR was 61.5 (Table 2). Regarding protection against A/H1N1/pdm09, before vaccination it was observed in only 5.6% of patients, however, the percentage of patients with protective anti-HAI titer increased significantly after immunization (to 41.8%) with the SR 38%; the GMTR ratio in this group was 8.35. More than 46% of vaccinated subjects seroconverted following vaccination regarding B Victoria lineage strain and the proportion of subjects with anti-HAI titer ≥ 1:40 was 57.4%; the mean fold increase was 10.3. A moderate response was observed for the B Yamagata lineage strain, i.e., seroconversion regarding vaccination was 48%; 17.6% of patients had protective anti-HAI titer before vaccination, this increased significantly (70.4%) after immunization. However, the mean fold increase was low (3.0).

### 3.4. Determinants of Seroconversion

Determinants of seroconversion after QIV vaccination by viral strain are presented in Table 3. Significant between-group differences were found with regards to seroconversion for B/Phuket/ 3073/2013 and BMI (*p* = 0.02), and influenza vaccination in the previous season/vaccination in life time (*p* = 0.03; *p* = 0.046). Similarly, for B/Colorado/06/2017 statistically significant differences were found in relation to proportions of patients who seroconverted and influenza vaccination in the previous season, as well as vaccination in life time (both: *p* < 0.0001). 

No significant between-group differences were found for any of 4 strains regarding sero-conversion after QIV vaccination and gender (*p* > 0.12), age (*p* > 0.33), alcohol uptake (*p* > 0.26), comorbidities (*p* > 0.23) and self-reported respiratory symptoms in the current season (*p* = 1.00). 

Proportions of participants with protective HAI titers before vaccination in the 2018/2019 season by the previous year vaccination are presented in Table 4. Only for B/Colorado/06/2017 strain the difference was significant (29.4% vs 4.4%, *p* = 0.005)

## 4. Discussion

### 4.1. Results Overview 

The results of this study showed a remarkably high GMTR after vaccination (61.5-fold) in the case of the A/H3N2/ strain. A much lower GMTR (3 to 10-fold) was observed regarding A/H1N1/ pdm09, B Victoria, and B Yamagata strains. About two-thirds of patients had post-QIV immunization protection for A/H3N2/ and B Yamagata vaccine strains; the lower rates (about 50%) were observed for A/H1N1/pdm09 and B Victoria. The SR was high for A/H3N2/ (64.8%) and relatively lower for B Yamagata (48.2%), B Victoria (46.8%) and A/H1N1/pdm09 (38%). Vaccination in the previous season significantly impaired the SR regarding both B strains.

### 4.2. Serologic Antibody Response after Influenza Vaccination 

In this study anti-HAI titers against A (A/H1N1/pdm09 and A/H3N2/), and B (Victoria and Yamagata) influenza viruses were low among unvaccinated individuals (GMT: 1.7, 1.0, 3.1, and 14.2, respectively). However, despite the weakening of numerous components of the immune system in the study group, due to the natural aging process [21,43], substantial antibody response following vaccination was observed. This referred to all four vaccine antigens, particularly to A/H3N2. Thus, adding a second B strain to a subunit, QIV did not compromise the immunogenicity induced by the other three strains. This outcome corresponds to the results of previous studies in elderly patients [29,30], including randomized controlled trials (RCTs) [16,36]. 

As an example, the results of phase III, randomized, double-blind, active-controlled, multi-center trial performed during the 2010/2011 influenza season in the US showed that - in adults ≥ 65 years of age - QIV induced non-inferior antibody titers compared with control TIVs for all four vaccine strains [36]. This finding is in line with the more current, similar randomized, multicenter trial conducted in the US in the same group of age, in the 2017 /2018 season [16]. The results showed that a quadrivalent high-dose (HD) vaccine-induced HAI antibody responses that were non-inferior to responses induced by a trivalent-HD vaccine for the three shared strains and superior HAI antibody titers for the additional B-lineage strain. 

In this study, a moderate response was observed for the B Yamagata lineage strain. The relatively low average post-vaccination increase in antibody titers (3.0) and seroconversion rate (47%) might be partly influenced by the fact that the same lineage strain was used in a QIV for the 2017/2018 season. Of note, about 16% of the study participants reported being vaccinated in the previous season with a QIV, which reflects the generally low uptake of influenza vaccines in Poland, especially in the elderly [11]. Previous exposure to influenza vaccine could have an impact on reduced antibody titers and SRs. Almost one in six patients showed protective antibody titers before vaccination, which increased significantly (70.4%) after immunization; QIV induced superior PR for the B Yamagata-lineage strain when compared with the other strains. 

The relative proportion of circulating influenza A/H1N1/pdm09 and influenza A/H3N2/ viruses in the European region varied by country in the 2018/2019 season. The proportion of influenza A viruses subtyped in patients from EU PCCs was ≥ 95%; about 60% were influenza A/H1N1/pdm09 viruses; however, this proportion was > 80% in Denmark, the UK and Poland [44].

Based on this mix of circulating influenza subtypes and variation within the antigenic likeness of circulating viruses with the egg-propagated vaccine component, vaccine effectiveness might vary across Europe [44]. Although, according to current research, the vaccine was less effective against A/H3N2/ influenza viruses in recent years [45], in the 2018/2019 season, the protection rate against the A/H3N2/ strain among elderly Polish patients from a PCC was higher than A/H1N1/pdm09 and influenza B Victoria. The same was noted in the A/H3N2/ strain and GMTR, showing over a sixty-fold increase after vaccination.

Until 2017/2018 season the WHO recommended TIV, and since 2018/2019 QIV have been recommended. From the 2017/2018 season the A/H1N1/pdm09 component, A/California/07/2009 - was replaced with the A/Michigan/45/2015 (H1N1) pdm09-like antigen. In the 2018/2019 season the A/H3N2/ component was changed from A/HongKong/4801/2014(H3N2) to A/Singapore/INFIMH- 16-0019/2016. For the Victoria lineage strain, B/ Brisbane/60/2008, was added to the vaccine in the 2017/2018 epidemic season, it was replaced in the 2018/2019 epidemic season with B/Colorado/06/ 2017. In 2013/2014 and 2014/2015 epidemic seasons B/Massachusetts/2/2012 (Yamagata lineage) was the vaccine strain, and this was replaced in the 2015/2016 epidemic season with the B/Phuket/ 3073/2013 [22,46,47,48]. 

In the current study, protection after QIV immunization, with an HAI antibody titer of ≥ 40, was found to be acceptable, particularly regarding A/H3N2/ and B viral strains; however, it was much lower for A/H1N1/pdm09. Interestingly, the high post-vaccination protection rate against A/H3N2/ was not the result of a high pre-protection rate, as none of the patients had protection before vaccination. Some previous observations also found that the A/H3N2/ vaccine antigen was able to induce a satisfactory immune response [49]. The reason for potential differences observed between studies in PRs and SRs could be related to the vaccine, the viruses or population exposure history [50].

Remarkably, the vast majority of the studied patients who had a QIV consisting of the same A/H1N1/pdm09 and B Yamagata antigens as in the previous season did not have protective antibody titers. This was also observed by Loebermann et al., who evaluated the immunogenicity of aTIV produced in mammalian cell culture administered to elderly adults. This may suggest that either antibody titers decline rapidly or that individuals did not develop a protective antibody titer earlier. Due to the fact that protective antibody titers from the QIV received the previous season could only be detected in a minority of immunized elderly patients, to recommend annual vaccination in this cohort, even if the antigen composition did not change from the previous season would be of value [51]. 

In the 2018/2019 influenza season, the B Yamagata vaccine strain had not been changed [22]. Therefore, one of the reasons for the relatively high pre-protection rate against B Yamagata (Table 4) might have been the long-term vaccine antigen stimuli. Another cause could be a pre-existing immunity derived from previous natural infection. However, the relatively low percentage of participants having protective HAI titers before vaccination in the 2018/2019 season in the group which had not been vaccinated previously indicates the first scenario is more likely.

The response rate to QIV antigens, measured by the percentage of participants showing at least a 4-fold increase in the HAI antibody titer after vaccination, as well as an average increase in antibody levels, was excellent regarding A/H3N2/, and relatively lower in the case of the other antigens. Therefore, formulation adjustments to increase the antigen concentration of the serotypes that have lower immunogenicity could increase effectiveness. It has been shown in the case of the elderly that for both, TIV and QIV, higher doses of antigens are associated with higher antibody responses to a vaccine [16,29].

### 4.3. Determinants of Serologic Antibody Response 

Repeated vaccination, as well as obesity, are well-known factors affecting the immune response after influenza vaccination [52,53,54]. 

In the case of influenza B, vaccination in the previous season, as well as vaccinations in the earlier seasons, had a negative impact on vaccine response among our participants; this was also observed by others [55,56,57]. As an example, Nebeshima et al. found that the HAI antibody titers to both influenza B strains in the repeated vaccination group of hospital workers were significantly lower than in the single vaccination group. This phenomenon had no relation to the pre-vaccination HAI titer, which suggested that the decreased HAI response to repeated influenza vaccination was mainly affected by the previous vaccination per se, rather than by the pre-existing antibody titer [57]. Similarly, to our findings, Sasaki et al. demonstrated that prior year vaccination was associated with sustained high HAI antibody titer one year on, but lower antibody response to the new vaccination [58]. In the current study vaccine responses were also impaired by pre-existing HAI titers regarding influenza B strains: 29.4% of patients vaccinated with a QIV in the previous season presented protective HAI-antibody titers for B Yamagata vs 15.4% not vaccinated patients; however, only 23.5% previously vaccinated patients seroconverted vs 55.9% in the not vaccinated group.

Human studies regarding obesity and its association with a decreased immune response to influenza vaccination have presented conflicting results [29]. The findings of the current study show that for B Yamagata strain significantly more normal weight patients seroconverted when compared to overweight and obese patients. This was also observed recently by Frasca et al. [59] who found reduced antibody responses to influenza vaccination in both young and elderly obese individuals. 

A correlation between health status in the elderly and HAI titers, with healthy older adults having significantly higher HAI titers after influenza vaccination than those with comorbidities was reported by other authors [29,43,60,61]. However, this was not observed in this study, possibly due to the relatively small sample size. Additional strategies that provide better protection of at-risk populations will be required to reinforce the efficacy of QIV in chronically ill elderly patients [29]. The immunization of family members, including children, and the vaccination of medical personnel, should be highly advocated.

### 4.4. Limitations

Several limitations exist in this study. Firstly, the sample size was relatively small, and power was limited. Therefore, the results obtained, particularly those from subgroup analyses, should be interpreted with caution. Secondly, information about the vaccination/infection history in the current season was obtained through a questionnaire, not from vaccination or medical records. Therefore, undocumented influenza exposure was likely, particularly among patients unvaccinated in the previous season, who presented a high pre-vaccination titer [60]. In addition, information reported as grams of pure alcohol consumption per week would be more instructive than the frequency of alcohol intake. However, to minimize potential measurement errors regarding cumulative measures of alcohol consumption, especially in the older age group, participants were queried on the frequency of alcohol intake. Other (unmeasured) factors could have also affected serologic response to QIV. Further studies on larger populations are needed to assess the response and its determinants better. Finally, only HAI titers were used to assess the immune response to vaccination. This might be challenging for influenza A/H3N2/, due to their fluctuating capacity to agglutinate red blood cells [34]. Consequently, to assess other measures of the immune response, such as anti-neuraminidase antibody levels or cell-mediated the immunity would be of great value.

## 5. Conclusions

Even though the influenza vaccination is reported to be less effective in the elderly compared to young individuals, partly due to decreased generation of specific serum antibodies and switched memory B cells [21,46], the QIV-induced antibody response in the study cohort was satisfactory. While the QIV vaccine had a tendency to work better against A/H3N2/ and influenza B viruses than A/H1N1/pdm09 in the elderly Polish population, this introductory vaccine immunogenicity study supports its use. 

The results show that a subunit QIV was immunogenic against the additional B lineage strain (B Victoria) without significantly compromising the immunogenicity of the other three vaccine strains. Adding a second B lineage strain in QIV could, therefore, provide broader protection against influenza B infection in this age group. As the QIV immunogenicity differed regarding the four antigens, formulation adjustments to increase the antigen concentration of the serotypes that have lower immunogenicity could increase effectiveness.

The study adds important data on the immunogenicity of influenza vaccines in Poland which have been lacking, even though QIVs have been available on the Polish market since the 2017/2019 season. These results should help encourage the switch to subunit QIV or other QIVs to protect Polish elderly patients against influenza.

The reduced response to immunization with influenza B strains in patients who had previously had influenza vaccinations, not consistent through the vaccine strains, needs further research to better understand influencing factors. 

## Figures and Tables

**Table 1:** Characteristic of study participants, Gryfino, 2018/2019; (n = 108).

Variable	N	%
**Age** [years] mean 66.7 (SD 6.7)		
55–67	59	54.6
68–85	49	45.4
**Gender**		
Female	49	45.4
Male	59	54.6
**BMI** [kg/m^2^] mean 28.1 (SD 4.9)		
<18.5	3	2.8
18.5–24.99	21	19.4
25.0–29.99	43	39.8
≥30.0	41	38.0
**Smoking**		
current	20	18.5
quit	45	41.7
never	43	39.8
**Alcohol Consumption**		
1–2 times a month	62	57.4
1–2 times a week	7	6.5
≥2 times a week/daily	3	2.8
non-drinker	18	16.7
had been drinking in the past	18	16.7
**Self-Reported Respiratory Symptoms in the Current Season**		
yes	14	13.0
no	90	83.3
did not know	4	3.7
**Comorbidities**		
yes	37	34.3
no	71	65.7
**Vaccinated in the Previous Season**		
yes	17	15.7
no	88	81.5
did not remember	3	2.8
**Vaccinated in Lifetime**		
never	68	63.0
once	15	13.9
>1	25	23.1

**Table 2:** Serologic antibody response after quadrivalent influenza vaccination by antigen used; Gryfino, Poland, 2018/2019 (n = 108).

Antigen	GMT ^1^		GMTR ^2^	PR (%) ^3^		SR (%) ^4^
	* Pre-	** Post-	** Post-	* Pre-	** Post-	* Post-
A/Michigan/45/2015 [A/H1N1/pdm09]	1.74	14.54	8.35	5.6	41.8	38.0
A/Singapore/INFIMH-16-0019/2016 [A/H3N2/]	1.00	61.53	61.53	0.0	64.8	64.8
B/Colorado/06/2017 [Victoria lineage]	3.08	31.76	10.29	8.3	57.4	46.8
B/Phuket/3073/2013 [Yamagata lineage]	14.18	43.02	3.03	17.6	70.4	48.2

^1^ Geometric mean of antibody titers; ^2^ Geometric mean titers ratio; ^3^ Protection rate—proportion of participants with HAI antibody titer ≥ 1:40; ^4^ Seroconvertion rate—proportion of participants with either (1) an HAI titer < 10 at day 0 and a post-vaccination (day 28–56) HAI titer ≥ 40 or (2) an HAI titer ≥ 10 at day 0 and a ≥4-fold increase in HAI titer between day 0 and post-vaccination; * Pre-vaccination; ** Post-vaccination.

**Table 3:** Determinants of seroconversion * after quadrivalent influenza vaccination by viral strain; n = 108, Gryfino, Poland, 2018/2019.

Variable		Viral Strain											
		A/Michigan/45/2015 ^a^			A/Singapore/INFIMH-16-0019/2016 ^b^			B/Colorado/06/2017 ^c^			B/Phuket/3073/2013 ^d^		
		n/N	%	*P*	n/N	%	*p*	n/N	%	*p*	n/N	%	*p*
Gender	Males	18/59	30.5	0.11	39/59	66.1	0.84	30/59	50.8	0.34	25/59	42.4	0.25
	Females	23/49	46.9		31/49	63.3		20/49	40.8		27/49	55.1	
Age (years)	55–67	25/59	42.4	0.32	37/59	62.7	0.69	25/59	42.4	0.44	29/59	49.15	0.85
	68–85	16/49	32.7		33/49	67.4		25/49	51.0		23/49	46.94	
BMI (kg/m^2^)	<25	10/24	41.7	0.81	13/24	54.2	0.23	15/24	62.5	0.10	17/24	70.8	**0.02**
	≥94	31/84	36.9		57/84	67.9		35/84	41.7		35/84	41.7	
Smoker	Current	9/20	45.0	0.61	13/20	65.0	1.00	11/20	55.0	0.46	14/20	70.0	0.05
	in the past/never	32/88	36.4		57/88	64.8		39/88	44.3		38/88	43.1	
Alcohol uptake	≤1–2/month	33/80	41.3	0.27	49/80	61.3	0.25	37/80	46.3	1.00	38/80	47.5	0.83
	>1–2/month	8/28	28.6		21/28	75.0		13/28	46.4		14/28	50.0	
Comorbidities	Yes	13/37	35.1	0.68	26/37	70.3	0.52	14/37	37.8	0.23	16/37	43.2	0.54
	No	28/71	39.4		44/71	62.0		36/71	50.7		36/71	50.7	
Self-reported respiratory symptoms in the current season	Yes	5/14	35.7	1.00	9/14	64.3	1.00	6/14	46.9	1.00	7/14	50.0	1.00
	No	36/94	38.3		61/94	64.9		44/94	46.8		45/94	47.9	
Vaccination ever in life time	Yes	11/40	27.5	0.10	21/40	52.5	0.06	5/40	12.5	< 0.0001 **	14/40	53.0	0.046 **
	No	30/68	44.1		49/68	72.152.5		45/68	66.2		38/68	55.9	
Vaccination in the previous season	Yes	3/17	17.6	0.05	10/17	58.8	0.38	2/17	11.8	< 0.0001 **	4/17	23.5	0.03 **
	No	30/68	44.1		49/68	72.1		45/68	66.2		38/68	55.9	

^a^ A/H1N1/pdm09 **^b^** A/H3N2/ **^c^** Victoria lineage **^d^** Yamagata lineage; * Seroconversion - pre-vaccination HI titer < 1:10 and a post vaccination HI titer > 1:40 or a pre-vaccination HI titer ≥ 1:10 and a minimum four-fold rise in post-vaccination HI antibody titer; ** statistically significant

**Table 4:** Participants with protective HAI antibody titers before vaccination in the 2018/2019 season by the previous season vaccination. Gryfino, Poland, 2018/2019 (n = 108).

Antigen	Not Vaccinated in the Previous Year n = 91		Previous Year Vaccination n = 17		*p*
	n	%	N	%	
A/Michigan/45/2015 [A/H1N1/pdm09]	5	5.5	1	5.9	1.00
A/Singapore/INFIMH-16-0019/2016 [A/H3N2/]	0	0.0	0	0.0	n.a.
B/Colorado/06/2017 [Victoria lineage]	4	4.4	5	29.4	0.005
B/Phuket/3073/2013 [Yamagata lineage]	14	15.4	5	29.4	0.18
